# What Does Immunology Have to Do With Normal Brain Development and the Pathophysiology Underlying Tourette Syndrome and Related Neuropsychiatric Disorders?

**DOI:** 10.3389/fneur.2020.567407

**Published:** 2020-09-16

**Authors:** Davide Martino, Isaac Johnson, James F. Leckman

**Affiliations:** ^1^Department of Clinical Neurosciences & Hotchkiss Brain Institute, University of Calgary, Calgary, AB, Canada; ^2^Child Study Center, Yale University, New Haven, CT, United States; ^3^Department of Psychiatry, University of California, San Francisco, San Francisco, CA, United States; ^4^Departments of Psychiatry, Pediatrics and Psychology, Yale University, New Haven, CT, United States

**Keywords:** Tourette, obsessive-compulsive disorder, attention deficit hyperactivity disorder, immunology, neuroinflammation, microbiome, microglia, neural organoids

## Abstract

**Objective:** The goal of this article is to review the past decade's literature and provide a critical commentary on the involvement of immunological mechanisms in normal brain development, as well as its role in the pathophysiology of Tourette syndrome, other Chronic tic disorders (CTD), and related neuropsychiatric disorders including Obsessive-compulsive disorder (OCD) and Attention deficit hyperactivity disorder (ADHD).

**Methods:** We conducted a literature search using the Medline/PubMed and EMBASE electronic databases to locate relevant articles and abstracts published between 2009 and 2020, using a comprehensive list of search terms related to immune mechanisms and the diseases of interest, including both clinical and animal model studies.

**Results:** The cellular and molecular processes that constitute our “immune system” are crucial to normal brain development and the formation and maintenance of neural circuits. It is also increasingly evident that innate and adaptive systemic immune pathways, as well as neuroinflammatory mechanisms, play an important role in the pathobiology of at least a subset of individuals with Tourette syndrome and related neuropsychiatric disorders In the conceptual framework of the holobiont theory, emerging evidence points also to the importance of the “microbiota-gut-brain axis” in the pathobiology of these neurodevelopmental disorders.

**Conclusions:** Neural development is an enormously complex and dynamic process. Immunological pathways are implicated in several early neurodevelopmental processes including the formation and refinement of neural circuits. Hyper-reactivity of systemic immune pathways and neuroinflammation may contribute to the natural fluctuations of the core behavioral features of CTD, OCD, and ADHD. There is still limited knowledge of the efficacy of direct and indirect (i.e., through environmental modifications) immune-modulatory interventions in the treatment of these disorders. Future research also needs to focus on the key molecular pathways through which dysbiosis of different tissue microbiota influence neuroimmune interactions in these disorders, and how microbiota modification could modify their natural history. It is also possible that valid biomarkers will emerge that will guide a more personalized approach to the treatment of these disorders.

## Introduction

Tourette syndrome (TS) is one of the most common neurodevelopmental disorders worldwide, with prevalence estimates ranging between 0.3 and 0.9% between 5 and 18 years of age (1, 2). TS is characterized by tics, i.e., patterned and recurrent, non-rhythmic movements and vocalizations that are partially suppressible with volition. The assessment and management of TS is profoundly influenced by the comorbidity (80–90% of patients) with other neurodevelopmental disorders (3), in particular obsessive-compulsive disorder (OCD) and attention deficit hyperactivity disorder (ADHD) (3). ADHD is the most common co-occurring disorder in TS (50–60% of cases), often pre-dating the onset of tics (4). ADHD comorbidity in TS is a major determinant of impairment of psychosocial and cognitive functioning, self-esteem and quality of life. Whereas, OCD coexists in 10–35% of TS patients, obsessive-compulsive symptoms (OCS) that do not reach, for severity and functional impairment, the threshold of “disorder” are present in up to 90% of TS patients (5, 6). Obsessions are recurrent and persistent thoughts that are unwanted, intrusive and distressing, whereas compulsions are elaborate, rigidly patterned voluntary actions or “mental acts” that may be responses to obsessions and/or executed to relieve a state of anxiety. Both the diagnosis of TS and the comorbidity with ADHD and OCD in the context of TS are more prevalent in males. In addition to comorbidity in the same individual, TS, OCD, and ADHD co-aggregate in families, with higher rates of both OCD/OCS and ADHD in first-degree relatives of TS patients (3).

**Figure 1:**
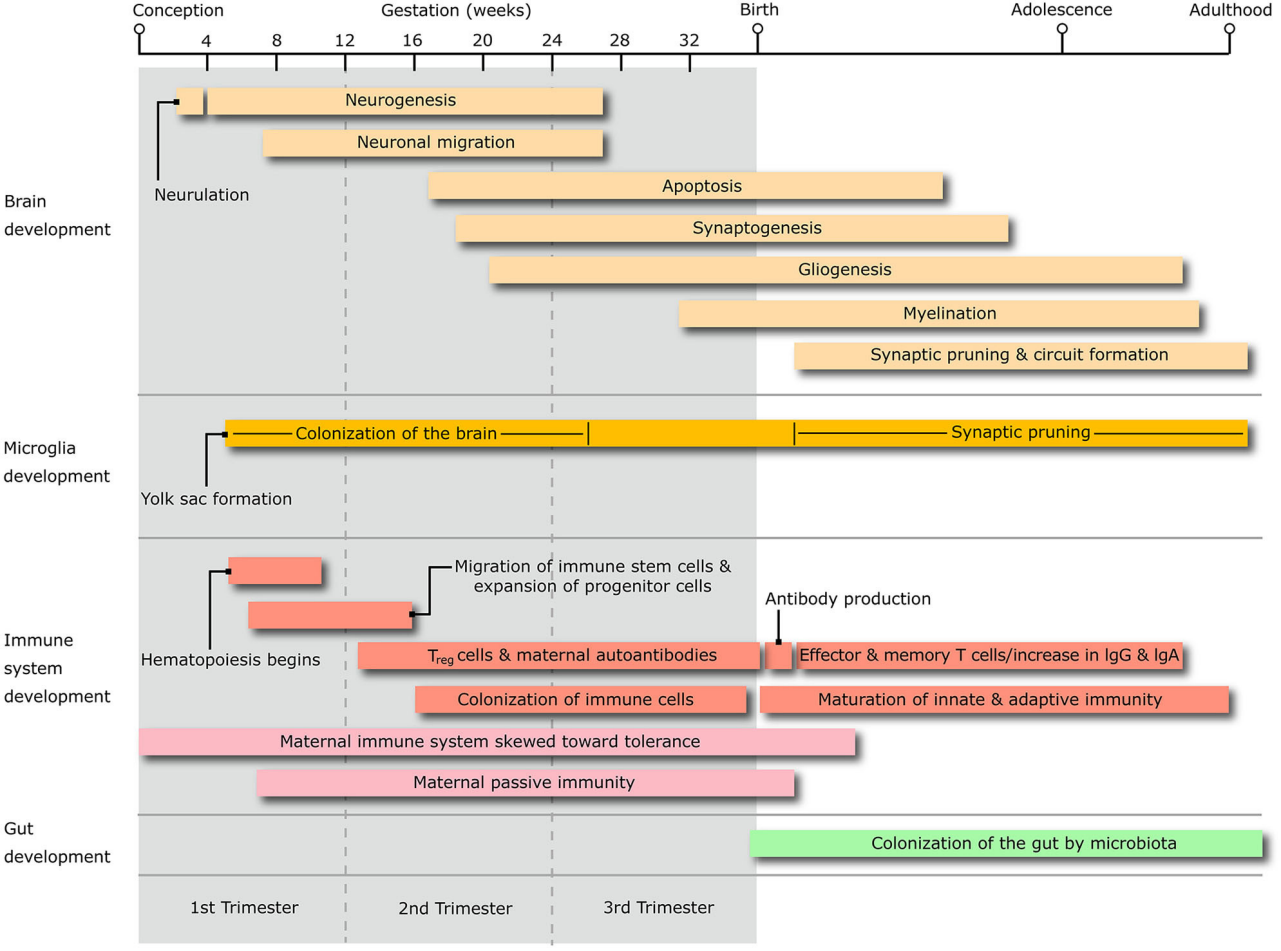
The diagram shows the timing of the salient stages of central nervous system development, alongside that of microglia, immune system and microbiota colonization of the gastrointestinal system, throughout the whole life span from conception to adulthood. T_reg_, T regulatory lymphocytes; IgG, immunoglobulin G; IgA, Immunoglobulin A.

In line with their familial aggregation, the comorbidity of OCD and ADHD in TS patients appears to be founded in part on genetic grounds. However, the genetic relationship amongst these disorders is complex, and potentially linked to specific sub-phenotypes, which may explain differences in the strength of their pairwise genetic correlation (7, 8). A recent study quantified the genetic sharing from genome-wide association studies across different neuropsychiatric conditions, yielding a significant genetic correlation between TS and OCD, but only a trend toward a correlation between TS and ADHD, and even weaker association between ADHD and OCD (9). Part of the reason for this different degree of genetic sharing is that extra-genetic and epigenetic factors may contribute to shared common pathophysiological mechanisms that support their comorbidity. It is believed that these pathogenic mechanisms ultimately generate abnormalities in the trajectory of maturation of sensory-motor and associative loops of the cortico-basal ganglia circuitry (10). Neuroimaging research in these conditions corroborated theories of accelerated and/or delayed maturation within cortico-basal ganglia and cortico-cortical pathways (11, 12). This abnormal connectivity may reflect anomalies of basic neurodevelopmental processes like synaptic formation and plastic refining, neurogenesis, and neuronal migration. Anomalies in these mechanisms may be induced, at least in part, by a dysfunctional neural-immune crosstalk that stems from problems in the maturation of innate and adaptive immunity [recently reviewed by (13)], in particular the colonization and maturation of immune cells that reside in the CNS, e.g., microglia (Figure 1).

Autism spectrum disorder (ASD) represents a “model” childhood-onset neurodevelopmental disorder in which genetic and environmental exposures in neural/immune development have been investigated during all stages (prenatal, perinatal, and early postnatal) of brain development. Maternal immune activation (MIA) rodent and non-human primate models of ASD helped elucidating the immune effects on neuronal gene dysregulation and behavioral phenotypes (14, 15). Human studies of ASD have generated hypotheses on the immune-modifying environmental exposures and immunological pathways potentially responsible for the atypical neural-immune crosstalk during different developmental stages (16). Genetic and environmental factors may, independently or interactively, contribute to a direct effect of cytokines and other immune effector molecules on neural cell progenitors, as well as to an abnormal trajectory of maturation of microglia, the multifunctional CNS-resident immune cell type (17).

MIA models have also been important in demonstrating a link between these processes and behavioral and cognitive phenotypes of human ASD (18) and, to a lesser extent, ADHD. Despite their comorbidity rate, though, advances on the immunobiology of TS, OCD, and ADHD have occurred at different pace, probably due to their difference in prevalence, phenotypic heterogeneity, and face and construct validity of representative animal models. Different immunological triggers of maternal-fetal and post-natal immune activation, e.g., infections, autoimmunity, stress, and microbiota constituents, have also been explored with different intensity across these three conditions. Moreover, most human studies exploring the “dysimmune” hypothesis in OCD and TS sought to identify parallel immunopathogenic mechanisms between these chronic, neurodevelopmental disorders and the distinct spectrum of pediatric acute neuropsychiatric syndromes (e.g., PANS), which manifest with OCS and other behavioral and cognitive features, including tics. The PANS spectrum differs from classical, neurodevelopmental OCD and TS in phenomenology, natural history, familial aggregation, prognosis and, probably, susceptibility to immune-based therapies, as extensively reviewed elsewhere (19–22). Therefore, whether mechanistic findings from animal and human studies of acute, putatively immune-mediated, forms of OCS, tics and ADHD-like symptoms can be translated as relevant to the pathophysiology of TS, OCD, and ADHD remains subject for debate.

**Figure 2:**
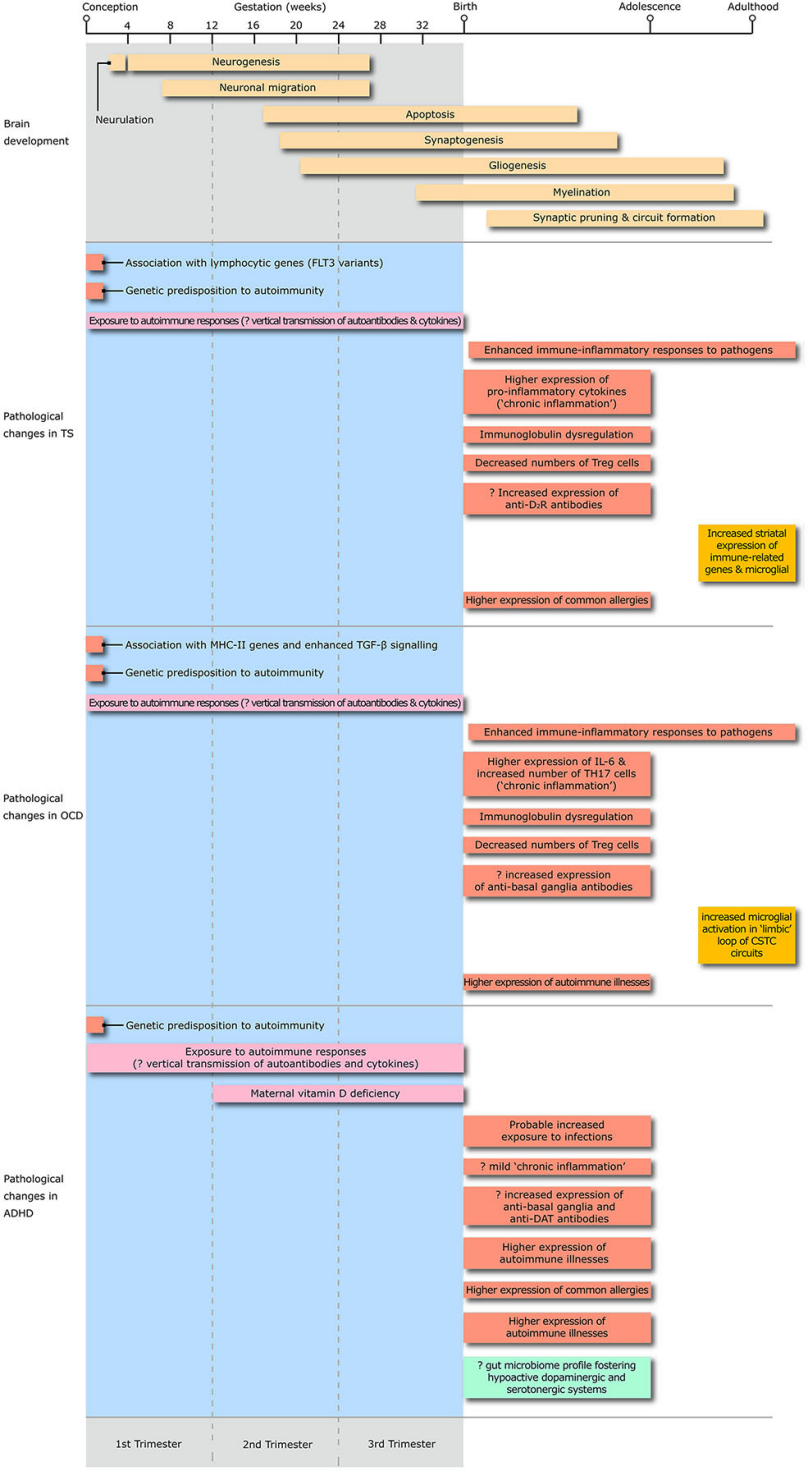
The diagram illustrates the the timing of exposure to risk factors for immune dysregulation, peripheral and central changes in immune-inflammatory responses, changes in microglial activation and alteration in the gut microbiome profile throughout the whole life span from conception to adulthood in Tourette syndrome, obsessive-compulsive disorder (OCD) and attention deficit hyperactivity disorder (ADHD). T_reg_, T regulatory lymphocytes; D2R, D2 dopamine receptor; IL, interleukin; TH17, T-helper 17 lymphocytes; CSTC, cortico-striato-thalamo-cortical; DAT, dopamine transporter.

The main objective of our review is to summarize the key advances in knowledge of risk factors, peripheral and CNS markers of immune dysregulation in TS, OCD, and ADHD (Figure 2). Topics include: their degree of comorbidity with immune-mediated illnesses, the contribution of representative model systems to the conceptualization of neural-immune crosstalk, and the pivotal mechanisms through which dysfunctional neural-immune crosstalk throughout development can contribute to the onset and natural history of these highly overlapping disorders.

## Risk Factors for Immune Dysregulation

### Genetic Predisposition

The genetic basis of immune dysregulation in immune-mediated diseases is known to be polygenic, supporting a differential expression of gene products functionally interacting within complex mechanistic pathways. Despite important knowledge advances regarding this “immunological” interactome for several autoimmune and neurodevelopmental illnesses (23, 24), this area remains under-explored and poorly understood in TS, OCD, and ADHD. The association of TS with genetic variants predisposing to immune dysregulation was explored by very few studies to date. A single study awaiting replication reported the association between tics and a single nucleotide polymorphism (SNP) of the *TNF* gene (−308 A/G) coding for the pro-inflammatory cytokine tumor necrosis factor (TNF-α), which controls its transcription and has been linked to atopic dermatitis, asthma, and Graves' disease (25). Summary-level data from genome-wide association studies (GWAS) demonstrated a positive genetic correlation between TS and allergy (26), which may contribute to the comorbidity between TS and allergic illnesses, reviewed below. A recent analysis of genome-wide data from 3,581 TS individuals and 7,682 ancestry-matched controls identified an association of TS with a Lymphocytic gene set, driven by variants in *FLT3* (27). This gene is critical for neuro-immune interactions, and its inhibition can alleviate peripheral neuropathic pain, a chronic neuro-immune condition.

A handful of studies produced interesting signals of an association between OCD and genes that are relevant to the development and control of the immune system. In particular, major histocompatibility class (MHC)-II molecules are crucial to the development and homeostasis of neural-immune crosstalk. An exon-focused GWAS identified MHC locus polymorphisms as top signals (28), whereas a case-control study comparing 144 early-onset OCD with general population samples found a significantly higher frequency of alleles comprising HLA-DRB1^*^04 in OCD (29). A Dutch population-based study (30) reported a significant association between OCD and *RFXANK*, a gene encoding another MHC-II protein, linked to an immunodeficiency disorder (bare lymphocyte syndrome 2). However, direct gene-gene interactions/pathways or gene-environment interactions relevant to OCD remain unknown. In the attempt to explore protein-protein interaction networks through the identification of *de novo* SNPs, Cappi et al. (31), highlighted enriched transforming growth factor-β (TGF-β) and glucocorticoid receptor signaling. TGF-β signaling controls immune homeostasis, direction of lymphocyte differentiation and aspects of embryonic development including neuronal migration and synapse formation. Two potentially high relevant genes highlighted by their analyses included *WWP1*, that codes for a protein inhibiting transcriptional activity induced by TGF-β, and *SMAD4*, coding for a signal transduction protein activated by TGF-β signaling during CNS proliferation and differentiation.

The evidence for genetic and epigenetic contribution to immune dysregulation in ADHD is also still preliminary. Independent genomic analyses showed an association between ADHD and pathways implicated in gene expression during development and immune-inflammatory regulation, which is shared with major depressive disorder (32, 33). The epidemiological association of ADHD and immune-mediated diseases is supported by correlational data from previous GWASs, that highlighted a modest genetic correlation with psoriasis, rheumatoid arthritis and susceptibility to ear infections and tuberculosis (26). An epigenome-wide association study (34) that meta-analyzed results from three ADHD cohorts revealed six differentially methylated regions in the MHC, including schizophrenia-associated *C4A* and *C4B* genes, from blood specimens of two cohorts. Larger, longitudinal studies should confirm these findings and exclude reverse causality (i.e., trait-induced DNA methylation variation). Finally, an exploratory gene expression regulation analysis through the expression of 13 microRNAs (35) found that all the differentially expressed genes in children with ADHD were involved in immune functions, including complement cascade and B-cell receptor signaling.

In summary, there is initial evidence of association with genetic variants modulating systemic immune regulation and immune mechanisms linked to neural development (e.g., TGF-β signaling) in OCD and ADHD. Evidence from GWASs also shows genetic correlation between TS, ADHD and several immune-mediated diseases.

### Pre- and Perinatal Exposures

Familial co-aggregation of TS, OCD, and ADHD with autoimmune diseases may indicate genetic predisposition to immune dysregulation, which, for maternal history, might be complicated by enhanced intrauterine immune-inflammatory mechanisms, e.g., vertical autoantibody transmission or dysregulated cytokine environment. A Swedish National Patient Register study (36) reported that mothers, fathers and siblings of individuals with TS were significantly more likely to have any autoimmune disorder, with risk increased by 40, 31, and 17%, respectively. They also found significantly increased risk of autoimmune diseases in first-degree relatives of OCD probands, with risk increased by 17% in mothers, 8% in fathers, and 16% in siblings. Recent anecdotal evidence suggests a link between maternal history of thyroid autoimmunity and an atypical, acute presentation of chronic neurodevelopmental disorders, including ASD, TS, and OCD (37). These associations need to be confirmed in larger clinical samples.

ADHD is also associated with increased frequency of maternal history of autoimmune illnesses and infections. A population study using Danish National Registers (38) identified a 12% increase of maternal, but not paternal, history of type 1 diabetes, autoimmune hepatitis, psoriasis, ankylosing spondylitis, and thyrotoxicosis. A stronger association was detected using longitudinal Norwegian registers (39), with increased risks ranging between 20% for maternal hypothyroidism and 80% for maternal multiple sclerosis, but not for non-immune conditions like hypertension. The association with hypothyroidism, however, might depend on maternal hypothyroxinemia rather than thyroid peroxidase antibodies (40).

Studies exploring the risk of ADHD in the offspring of women with gestational infections yielded noteworthy methodological heterogeneity and inconsistent results. Following reports (41, 42) of an association with viral rash or respiratory infections, Mann and McDermott (43) and Silva et al. (44) reported a 29–37% increase in risk of ADHD in the offspring of women with gestational bacterial or fungal genitourinary infection using record-linkage datasets. Evidence from the Danish National Birth Cohort reported an increase in risk associated with genitourinary infection (33–60%) from the third to the eighth month of pregnancy (45). However, sibling-comparison analyses conducted using Swedish National Registers (46) suggested that this association could spuriously result from unmeasured familial confounding. Similar findings came from a Canadian population-based retrospective cohort study that used sibling-comparison analyses to demonstrate lack of association between prenatal antibiotic exposure and ADHD risk ADHD in children (47).

An association of maternal vitamin D deficiency—predisposing to immune dysregulation- and ADHD in offspring was documented by population birth cohorts. A study pooling five Spanish birth cohorts (48) showed that ADHD severity in the offspring at ages 4–5 decreased by 11% for the inattention scale and by 12% for the hyperactivity-impulsivity scale per 10 ng/ml increment of 25(OH)D3 plasma concentration at 13 weeks of gestation. A subsequent population study using Finnish registers (49) detected a 53% increase of ADHD risk in the offspring of women at the highest quintile of maternal 25(OH)D levels compared to those at the lowest quintile. Maternal C-reactive protein serum levels during early gestation were, instead, not associated with risk of ADHD in the offspring (50).

Cumulatively, familial co-aggregation of autoimmunity may influence genetic and prenatal risk of TS, OCD, and ADHD in the offspring. The higher risk associated with maternal autoimmunity and vitamin D deficiency in pregnancy suggests an additive or multiplicative effect of autoimmunity through interaction of genetic and vertical risk transmission. However, the questionable association between gestational genitourinary infections and ADHD in offspring highlights the risk of confounding from unobserved factors shared within families in population studies.

### Postnatal Exposures

The influence of immunogenic triggers, primarily infections and stress, on the development and clinical course of TS, OCD, and ADHD is incompletely understood. A body of evidence supports the enhancement of immune-inflammatory responses toward common pathogens in TS and OCD. The occurrence of tics in acute neuropsychiatric syndromes associated with Group A streptococcus (GAS) pharyngotonsillitis offered a rationale to cross-sectional clinical studies that revealed stronger anti-streptococcal antibody responses in these patients across different ages (51–54). Retrospective population cohort data from US and Denmark support a 35–59% increase in risk for a diagnosis of tic disorders and GAS exposure in the past year (55–57), although one similar study from the UK did not confirm this association (58). A similar rise in antibody responses was observed for obligate intracellular bacteria like *Chlamydia trachomatis* and *Mycoplasma pneumoniae* (59). Population data from Taiwan showed a 24% increase in risk of tic incidence in individuals with non-CNS enterovirus infection (60). Danish health registers data indicated that individuals with a non-streptococcal throat infection have a 25% increased risk of tic disorders, whereas those requiring hospitalization for infection and anti-infective treatment manifest a higher than 300% increase (57, 61). This evidence demonstrates greater risk of different common infections in individuals who will go on to develop TS, even if it cannot be considered proof of causative relationship. On the other hand, there is no evidence from clinic-based data supporting a temporal association between onset or clinical worsening of tics and a recent exposure to GAS in the context of TS (62–64). A recent prospective cohort study of 715 children with TS from different European countries did not detect any association between recent GAS exposure and clinically relevant exacerbations of tics (65).

Healthcare population registries provide some support to an association between OCD and prior infections, particularly with GAS pharyngeal infections. A GAS throat infection in the year prior to symptom onset was associated with a 76% increase in risk of developing OCD in a US health insurance claims database (56), but this finding was not confirmed by UK record-linkage primary care data (58). A more recent study of multiple Danish national healthcare registries (57) reported that a previous record of one or more positive rapid antigen diagnostic tests for GAS was associated with a 51% significantly higher risk of OCD diagnosis, independent on age at first positive test, compared to individuals without a streptococcal test. Importantly, individuals with a non-streptococcal throat infection also had a 28% significantly higher OCD risk. As with TS, smaller prospective clinic-based studies failed to show an association between exacerbation of OCS severity and recent GAS throat infections (62, 63, 66). None of the prospective clinic-based studies that explored the association between GAS exposure and changes in tic or OCS severity analyzed with accuracy the specific risk of different modalities of exposure (“colonization” vs. “infection”). This notwithstanding, the negative results of the largest of these studies focusing on tic severity (65) suggest that any association between GAS exposure and at least tic exacerbations is unlikely.

As for TS, an association of OCD with other pathogens cannot be excluded. Apart from case reports of concurrent *Mycoplasma pneumoniae* or varicella zoster virus infections (67, 68) in OCD, an association with a 2.5 to 4-fold increased seroprevalence of antibodies to *T. gondii* (69) has been reported (70, 71). At best, however, this anecdotal evidence suggests a proneness to comorbidity with a broad range of infections, similar to what observed for TS. There is also limited knowledge on whether the characteristics of tics and OCS may differ depending on prior/current exposure to infections. A recent report showed that young OCD patients with higher frequency of self-reported ear or throat infections have increased severity of cleaning/contamination-related symptoms (72), although an altered perception of their medical history and subsequent over-reporting might also explain this finding.

Psychosocial stressors predict short-term future severity of tics, OCS and depressive symptoms in TS, and have an established mechanistic link with immune-inflammatory responses. A clinic-based prospective study observed a multiplicative interaction between psychosocial stress and GAS infections as predictors of future tic severity (73). Immune-inflammatory responses to infections and activation of the hypothalamus-pituitary-adrenal (HPA) axis during stress responses might influence each other, contributing to tic exacerbation, but the neurobiological basis of this process needs to be elucidated (74).

Very pre-term infants exhibit an increased risk of ADHD symptoms during childhood following exposure to neonatal infections (75) and systemic inflammation, the latter expressed by high concentrations of neurotrophic proteins (76). Service- and population-based studies suggested an increase in future ADHD symptomatology in children with prior history of bacterial meningitis (77) and Enterovirus encephalitis (78), although the possibility of neuropsychological sequelae following direct neural tissue insult is high. ADHD behavioral patterns might contribute to increased chances of contact with pathogens (79), especially those with intrafamilial spread like polyomaviruses (80). Scandinavian national register studies provide the strongest evidence for increased risk of ADHD outcome in children exposed to infections requiring hospitalization [by 109% in (61)] and/or anti-infective treatment [by 56% in (61), and 10–60% for antibiotic exposure throughout the first 2 years of life in (81)]. Overall, there is supportive evidence of an association between prior infectious exposures in early childhood and ADHD diagnosis or symptoms, although these epidemiological studies do not yet constitute a clear proof of causality.

## Immune-Inflammatory Markers (see Table 1)

### TS

Even with some discrepancies, peripheral immune responses in TS are skewed toward pro-inflammatory mechanisms in most cross-sectional observational studies. Longitudinal observation showed covariation of TNF-α and interleukin (IL)-12 plasma levels and tic/OCS severity (82), regardless of medical treatment or concurrent infections. A subsequent study reported decreased TNF-α and soluble IL-1 receptor antagonist levels (83). A positive correlation between tic severity and IL-2 levels was reported in one study (84), whereas another reported increased IL-2 and IL-12 circulating levels only in TS patients with comorbid OCD (85). Further reports documented higher circulating levels of IL-6, IL-1β, IL-17 (86), and neopterin, a pteridine synthesized by the monocyte-macrophage cell lineage and linked to cell-mediated T_H_1 pro-inflammatory responses (62, 87).

**Table 1:** Summary of findings on peripheral and central inflammatory markers in Tourette syndrome, obsessive-compulsive disorder and attention deficit hyperactivity disorder.

**Inflammatory markers**	**Tourette syndrome**	**Obsessive-compulsive disorder**	**Attention deficit hyperactivity disorder**
**Peripheral**			
Dysregulated innate immune responses	• Lower TLR4 expression^#^ • Lower concentrations of TNF-α and soluble IL-1 receptor antagonist (*pooled age groups including adults*)^#^ • Higher concentrations of soluble CD14^#^ • Lower concentrations of IgG3 and IgA^#^	• Lower concentrations of IgA (only in male patients)^*^ • Lower vitamin D levels (inverse correlation with severity of obsessions)	
Dys(hyper)-regulated cell-mediated pro-inflammatory responses (“chronic” inflammation)	• Higher concentrations of IL-2, IL-6, IL-1β, IL-17*^#^* • Covariation of TNF-α and IL-12 plasma concentrations and tic/OCS severity*^#^* • Correlation of IL-2 concentrations with tic severity*^#^* • Higher concentrations of neopterin*^#^* • Higher concentrations of IL-1β, MCP-1 and IP-10 in patients with acute exacerbation of tics requiring hospitalization*^#^*	• Higher concentration and *ex vivo* production of IL-6 (moderating effect of comorbid major depressive disorder; *mostly from studies in adults*)^¶^ • Higher concentration of IL-1β (only in drug-naïve patients; *mostly from studies in adults*)^¶^ • Higher concentration of IL-2, IL-8, IL-17, soluble TNFR 1 and 2, CCL3 and CXCL8 (*adults*)*^#^* • Covariation of TNF-α and IL-12 plasma levels and tic/OCS severity*^#^* • Higher concentration of IL-1β, MCP-1 and IP-10 in patients with acute exacerbation of OCS requiring hospitalization*^#^*	• Higher concentration of IL-6^*^ • Correlation of executive function performance with IL-16 and IL-13 plasma levels^*^
Altered distribution of immune cell population	• Decreased numbers of CD4+CD25+ T-cells (Tregs)*^#^*	• Decreased numbers of CD4+CD25+ T-cells (Tregs) • Increased numbers of T_H_17 lymphocytes (Both changes associated with disease duration and severity)	
Altered immune cell gene expression	• Over-expression of genes related to pathogen recognition mechanisms and NK and CD8+ T-cell activation*^#^* • Correlation of genes controlling signaling pathways of immune-modulating neurotransmitters (GABA, acetylcholine and catecholamines) and tic severity*^#^*		
Autoantibody production	• Probable association between anti-D_2_R antibodies and clinically relevant exacerbation of tic severity*^#^*	• 5-fold greater proportion of seropositivity to antibodies targeting basal ganglia neural tissue (*pooled age groups including adults*)^¶^	• Probable association with anti-Yo antibodies*^#^* • Anti-DAT antibody titers higher in children carrying two 10-repeat alleles of the *DAT* gene; trend toward normalization after 1–2 years of treatment with methylphenidate*^#^*
**Central**			
	• Up-regulation of an immune-related gene module involved in the activation of microglia and increased number of CD45-positive cells and local microglial reaction within caudate/putamen (*postmortem from adult brains*)	• 31–36% higher TSPO-V_T_ in the “limbic” loop of the cortico-striato-thalamo-cortical circuitry (dorsal caudate/putamen, orbitofrontal cortex, thalamus, and ventral striatum) (PET *in adults*)	

*All findings are referred to children or adolescents unless indicated otherwise*.

* Population-based studies.

# *Clinic-based studies*.

¶ *Meta-analysis of studies with different design*.

*TLR, toll-like receptor; Ig, immunoglobulin; TNF, tumor necrosis factor; CD, cluster of differentiation; IL, interleukin; TNFR, tumor necrosis factor receptor; OCS, obsessive-compulsive symptoms; CCL3, Chemokine (C-C motif) ligand 3; CXCL8, chemokine (C-X-C motif) ligand 8; MCP-1, Monocyte Chemoattractant Protein-1; IP10, Interferon gamma-induced protein 10; T_H_, T-helper; NK, Natural Killer; GABA, gamma-Aminobutyric acid; D_2_R, dopamine D_2_ receptor; DAT, dopamine transporter; TSPO-V_T_, translocator protein distribution volume; PET, positron emission tomography*.

An increased susceptibility to infections and, perhaps, autoimmune processes might be supported by defective immune mechanisms (innate and adaptive) that protect against infections and autoimmunity under physiological conditions. Weidinger et al. (88) documented an impairment of innate responses in a cross-sectional study that compared 33 TS patients to 31 healthy subjects, showing lower receptor expression of toll-like receptor 4 (TLR4) after stimulation with lipopolysaccharide (LPS), and higher levels of soluble Cluster Differentiation (CD)14. These results could indicate inhibition of monocyte responses to LPS, suggesting defective innate responses to pathogens that would activate a vicious cycle of increased susceptibility to infections and sustain chronic inflammation. Immunoglobulin (Ig) synthesis may be dysregulated (reduced IgG3 plasma level) in TS patients (84). This Ig subclass promotes the classical complement cascade and pathogen elimination; hence, its downregulation might be related to higher infection exposure and overactive systemic inflammation. Finally, overactivation of immune responses in TS may be facilitated by lower numbers of CD4+CD25+ T-cells (Treg) in the periphery (89). Treg cells develop during physiological T-cell maturation in the thymus, survive in the periphery for constant monitoring of self-antigens, and prevent autoimmune responses.

Transcriptomic studies on peripheral blood mononuclear cells revealed different gene expression patterns throughout different developmental periods, with TS patients over-expressing genes related to pathogen recognition mechanisms before age 9, and over-expressing genes involved in Natural Killer (NK) and CD8+ T-cell activation during puberty. Furthermore, tic severity correlated with the expression of genes that control signaling pathways of immune-modulating neurotransmitters involved in tic generation in the CNS, like GABA, acetylcholine, and catecholamines (90–92). Increased expression of the *DRD5* gene in lymphocytes might contribute to decreased Treg numbers (93). However, these gene expression studies were performed on small clinical samples and provided correlational, but not causative, evidence.

Dysregulated systemic immune responses can promote the development of autoimmunity. However, the nature and severity of autoimmune processes in TS, and their role in the pathogenesis of tic disorders, remains unclear. Earlier investigations of autoantibodies in TS have used antigen-denaturing methods (94, 95) or immunoprecipitation techniques (96) that may detect non-specific antibody reactivities, or indirect immunofluorescence that does not allow the characterization of the target antigens. Independent reports using denaturing techniques could not demonstrate a significant titer rise of antibodies proposed as relevant in putative post-streptococcal disorders like Sydenham's chorea (97, 98).

Qualitative and quantitative measurement of IgG cell surface binding to live cells expressing candidate self-antigens is much more accurate to detect antigen-antibody reactivities occurring *in vivo*. Earlier studies applying immunofluorescence-based live-cell assays to explore circulating antibodies in TS patients demonstrated antibodies binding to the surface of rat striatal neurons or neuroblastoma cell lines (99, 100). This finding, however, was not replicated by a subsequent study that applied fluorescence-activated cell sorting (FACS) to live differentiated cells from the SH-SY5Y cell line (101). Dale et al. (102) demonstrated the presence of anti-D_2_ dopamine receptor (D_2_R) antibodies in the serum of 4 of 44 TS patients, using FACS applied on human embryonic kidney (HEK) cells transfected with a commercial form of the D_2_R; none of these 44 patients were positive for anti-D_1_ dopamine receptor antibodies. Very recently, Addabbo et al. (103) tested anti-D_2_R antibody reactivity in sera from 137 children with TS at a baseline time point and at time points coinciding with and following a clinically relevant tic exacerbation. These authors used a similar protocol to the one used by Dale et al. (102), but adopting a transfection method on Chinese Hamster Ovary (CHO)-K1 cells (102). Anti-D_2_R antibodies were detected in 6.6% of patients at baseline; an additional 9.8% of patients seroconverted during the peri-exacerbation time period, independently from new concurrent GAS infections. Although this does not demonstrate a causal link between anti-D_2_R antibodies and tics, it supports the implication of autoimmunity and concurrent behavioral-immunological fluctuations in TS. An earlier cross-sectional report on 51 patients with TS did not detect any specific serum autoantibodies associated with encephalopathies (targeting LGI1, CASPR2, NMDAR, AMPA1/2, GABAB1/B2) (104). More recently, Baglioni et al. (105) investigated the same patients tested by Addabbo et al. (103) plus a cohort of unaffected siblings of these patients, screening for autoantibodies associated with established encephalopathies (antibodies targeting NMDAR, CASPR2, LGI1, AMPAR, and GABAAR). Using live cell-based assays applied to live rat hippocampal neurons, only two individuals (one patient, one sibling) tested weakly positive for anti-NMDAR antibodies, indicating unlikely association with these pathogenic antibodies. Another report from the Hannover group applied indirect immunofluorescence to HEK cells to confirm the absence of these autoantibodies in the cerebrospinal fluid (CSF) of 20 adults with TS (106). Due to the relative paucity of *postmortem* data, the presence of autoantibodies in the brain of TS patients has not been systematically explored. Moreover, unlike for autism, the presence of circulating serum anti-fetal brain antibodies has not been investigated in mothers of children with TS.

There is initial evidence of enduring inflammatory changes in the neural tissue of patients with TS. Neuroinflammatory patterns may reveal direct and indirect links between immune-mediated processes and altered neuronal/synaptic maturation and functioning. Earlier studies (107, 108) that adopted a microarray transcriptomics approach on very small samples of adult TS *postmortem* brains documented a significant putaminal increase in the expression of genes coding for IL-2, IL-2 receptor β, and monocyte chemotactic factor-1. These results seemed to suggest a transcriptomics pattern in keeping with signaling pathways promoting T-cell and microglial proliferation and activation, increased blood-brain barrier permeability, and neural stem cell differentiation.

A subsequent RNA sequencing transcriptomics study of *post mortem* tissue from the caudate/putamen of nine TS subjects and nine age- and sex-matched control individuals [5 males and 4 females per group; (109)] provided more robust evidence of microglial involvement in this condition. These authors detected 309 down-regulated and 822 up-regulated genes, and 17 gene co-expression modules, identified with data-driven gene network analysis, in the striatum of TS brains. The top-scoring up-regulated module included immune-related genes involved in the activation of microglia. Consistent with this, they observed an increase in CD45-positive cells and local microglial reaction within caudate/putamen. This up-regulated “immune-related” module did not overlap with “neuronal” and “astrocyte” modules comprising striatal interneuron-related transcripts and transcripts related to cell adhesion and astrocyte-related metabolic pathways, respectively. The limited sample size did not allow the exploration of sex-dependent effects on the association between “immune-related” gene expression and TS. Even considering the long interval between onset and specimen collection, this finding suggests that immune and neuronal events co-occur, in part independently, in the brain of TS patients. It also provides preliminary, but compelling, evidence that the expression of molecular pathways associated with microglial maturation and functioning is involved in TS, adding this disorder to the spectrum of neurodevelopmental disorders linked to microglial dysfunction.

### OCD

The vast majority of studies investigating peripheral immunity in OCD focused on cross-sectional adult clinical samples, with large heterogeneity across studies. Therefore, an association between specific immune effector molecules and OCD remains unconfirmed. Meta-analytic estimates are available for the serum levels of six different cytokines that were explored in 31 studies (110). Similar to TS, the existing studies provide modest support to the overactivity of pro-inflammatory mechanisms. However, the heterogeneity of study populations and the intrinsic complexity of the OCD spectrum across different age groups indicate that immune dysregulation can be detected only after adjustment for potential confounding variables, a consideration that could be applied also to studies of TS patients. For example, co-morbid major depression moderates the association between OCD and raised IL-6 serum levels. Increased *ex vivo* production of IL-6 by LPS-stimulated macrophages could be detected including exclusively studies that enrolled adult patients without other behavioral comorbidities and gender- and age-matched control participants. The exposure to medications like SSRIs could also confound the association between OCD and cytokine levels: for instance, a significant increase in IL-1β serum levels was observed only including in the meta-analysis studies that enrolled drug-naïve patients. Meta-analyses and more recent observational studies (111) related to serum levels of other cytokines (TNFα, IL-4, IL-10, interferon [IFN]-γ) failed to demonstrate an association with OCD. The same study observed correlations with specific executive function performances in the OCD patient group. Finally, other cytokines were explored only in isolated or smaller studies, and therefore require replication. These studies reported raised serum levels of IL-2, IL-8, IL-17, soluble TNF receptors 1 and 2, and chemokines like CCL3 and CXCL8. Like for TS, these findings are overall consistent with a possible state of low-grade inflammation and propensity to exacerbate after exposure to appropriate triggers, e.g., infections. Acute exacerbation of OCD and tics requiring hospitalization was also associated with raised peripheral levels of IL-1β, MCP-1, and IP-10 (112).

A predisposition to autoimmune and post-infectious organ-specific immune responses could be related to IgA deficiency and altered distribution of different immune cell types. A retrospective study using electronic health records of 206 children and 1,024 adults with OCD showed that pediatric OCD patients have a 93–98% greater chance of manifesting serum IgA deficiency than children with ASD and anxiety disorders, but not higher than children with TS, and comparable to that of children diagnosed with celiac disease. Interestingly, this difference was significant only in males (113). Another controlled clinic-based study on 99 youth with OCD and 46 healthy volunteers showed higher frequencies of T-helper (Th)17 cells and lower percentages of Tregs in OCD (114). These changes became more marked as disease duration and severity increased and were independent of the comorbidity profile or exposure to psychoactive substances, suggesting the possibility of progressive immune dysregulation in OCD. Longitudinal studies should confirm whether immune dysregulation has indeed a dynamic pattern in this condition. Interestingly, autoreactive Th17 cells triggered grooming behaviors in a mouse model of experimental autoimmune encephalomyelitis (115). An isolated report identified lower vitamin D levels and inverse correlation between 25OH-D3 and severity of obsessive thoughts (116).

The characterization of Pediatric Autoimmune Neuropsychiatric Associated with Streptococcal Infection PANDAS; (117) has fostered the exploration of immunopathogenic mechanisms involving autoimmune processes and the role of autoantibodies (100, 118, 119) in the whole spectrum of OCD. These antibodies target D_1_ and D_2_ dopamine receptors, tubulin (120), and lysoganglioside (121), and were found to activate calcium calmodulin-dependent protein kinase II (CaMKII) in a SKNSH human neuronal cell line (121). These reactivities were proposed as markers of acute post-streptococcal OCS, targeting dopaminergic neurotransmission within the cortico-basal ganglia circuitry (122, 123). However, the reliability of this autoimmune panel (aka Cunningham panel) is debated, and the relevance of these autoantibodies to chronic OCD remains to be demonstrated. Initial data indicate that antibodies targeting striatal cholinergic interneurons may also play a role in PANDAS pathogenesis (19, 124).

Antineuronal antibodies were explored also in youth and adults with “garden variety” OCD. Earlier studies applying denaturing protocols of enzyme-linked immunosorbent assay and Western immunoblotting reported discrepant results on antibody reactivities to basal ganglia or other human brain tissue specimens from serum or CSF samples of patients with typical OCD (125–128). Other reports showed greater anti-brain antibody reactivity only in OCS in the context of PANDAS, but not in other children with OCD, compared to healthy subjects (129, 130). In an attempt to resolve this discrepancy, a systematic review, and meta-analysis of seven case-control studies cumulatively exploring 844 participants with primary OCD reported a significantly 5-fold greater proportion of seropositivity to antibodies targeting basal ganglia tissue compared to different control groups, and independent of demographics, disease characteristics, immunostaining method, study quality, publication type or publication bias, and sample size (131). The results of this meta-analysis still need to be taken with caution, given that there was a substantial variability of the accuracy of the different immunostaining assays used across the selected studies. Importantly, it remains undemonstrated whether the antigenic targets of these anti-basal ganglia antibodies are expressed on the cell surface and could therefore be central to a pathological mechanism affecting neuronal cells.

Similar to TS, the direct evidence of brain inflammatory changes in OCD is extremely limited. The link between a peripheral low inflammation state and neuroinflammation remains undemonstrated, as two independent studies that measured cytokine levels in the CSF of OCD patients reported discrepant results. To the best of our knowledge, evidence of activation of inflammatory pathways, either direct (based on immunohistochemistry or cytopathology) or indirect (based on transcriptomics) is lacking from *postmortem* brain tissue of OCD patients. The only evidence in this respect came from a positron emission tomography (PET) study that used a second-generation radiotracer binding the translocator protein TSPO (132). The density of this protein on microglia increases following activation and is expressed by its distribution volume (V_T_). Twenty OCD patients in their third decade of age (11 women) and 20 age-matched healthy volunteers were compared in TSPO-V_T_ using the *N*-(2-(2-fluoroethoxy) benzyl)-*N*-(4-phenoxypyridin-3-yl) acetamide ([^18^F]FEPPA) PET tracer. The SNP rs6971 of the *TSPO* gene was analyzed as a nuisance factor because it may influence the binding to this radiotracer (high-affinity homozygotes vs. mixed-affinity binding heterozygotes). TSPO-V_T_ was significantly 31–36% higher in OCD patients throughout the whole “limbic” loop of the cortico-striato-thalamo-cortical circuitry, i.e., in the dorsal caudate/putamen, orbitofrontal cortex, thalamus, and ventral striatum. Milder increases were reported in the anterior cingulate, medial prefrontal, ventrolateral prefrontal, insular, temporal cortical regions, and hippocampus. Interestingly, TSPO-V_T_ in the orbitofrontal cortex significantly positively correlated with greater distress associated with preventing compulsive behaviors. These findings provide the first direct evidence of neuroinflammatory changes in the brain circuits associated with OCD and indicate that activated microglia (and related potentially harmful M1 responses) may be present in adults with this condition, even many years following onset. This study obviously does not indicate a causative link, but rather a strong evidence of correlation, between elevated TSPO and OCD, which needs to be taken with caution also due to the other potential cellular pathomechanisms associated with TSPO elevation (e.g., cholesterol translocation from outer to inner mitochondrial membranes).

### ADHD

Despite the observed association with immune-mediated illnesses, there has been limited research on systemic immune regulatory mechanisms in ADHD. Most reports showed lack of significant differences of circulating levels of pro- and anti-inflammatory cytokines, as well as of other chronic immune activation markers, e.g., markers of the tryptophan-kynurenine pathway, of neurotrophic markers of glial integrity, e.g., S100-B protein, and of immunomodulating molecules like vitamin D3 in both pediatric (133–137) and adult (138, 139) populations. The only cytokine reported as significantly increased in ADHD in two independent studies has been IL-6 (140, 141). At the same time, the observation that raised circulating concentrations of pro-inflammatory cytokines, including IL-6, in the first 2 weeks of life of preterm infants are associated with attention difficulties at 2 years of age (142), and the correlation of executive function performance with IL-16 and IL-13 plasma levels in ADHD children (134), suggests an influence of mild chronic inflammation on the development of prefronto-subcortical connections related to attention and executive functions (143).

Autoantibody markers in ADHD have been investigated by a handful of studies during the past decade. Two studies reported higher immunoreactivity for anti-Purkinje cell antibodies in ADHD (144, 145). The association of ADHD with antibodies against basal ganglia homogenate (146) and dopamine transporter [DAT; (147)] was reported only by isolated studies. Basal anti-DAT antibody titers were higher in ADHD children carrying two 10-repeat alleles of the *DAT* gene, and tended to normalization after 1–2 years of treatment with methylphenidate. The presence of high serum anti-DAT antibodies supports hypermethylation at CpG1 position on the *DAT* gene as an epigenetic marker of ADHD severity (148).

Overall, the evidence of enhanced immune-inflammatory mechanisms in ADHD provides modest support in favor of overactive systemic immune mechanisms only in younger patients. Finally, an important knowledge gap is characterized by the complete lack of studies directly evaluating CNS inflammatory changes in ADHD.

## Comorbidity With Immune-Mediated Illnesses (Table 2)

### TS

Whereas, secondary tics may occur in autoimmune conditions like Sydenham's (or rheumatic) chorea, a systematic review by Perez-Vigil et al. (149) highlighted the dearth of investigations on autoimmune comorbidities in TS. A population-based investigation using the Swedish National Patient Register that assessed the association between TS and 40 autoimmune diseases (36) observed increased risk for comorbidity with Hashimoto's thyroiditis, celiac disease, scarlet fever, type 1 diabetes mellitus, and psoriasis (Table 2).

**Table 2:** Summary of findings on immunological comorbidity in Tourette syndrome, obsessive-compulsive disorder and attention deficit hyperactivity disorder.

**Comorbidity**	**Tourette syndrome**	**Obsessive-compulsive disorder**	**Attention deficit hyperactivity disorder**
**Autoimmune diseases** (% refer to the increase in risk of diagnosis of the specific autoimmune disease)	Hashimoto's thyroiditis (106%)^*^ Celiac disease (67%)^*^ Scarlet fever (62%)^*^ T1DM (37%)^*^ Psoriasis (33%)^*^	Sjögren's syndrome (94%)^*^ Celiac disease (76%)^*^ Guillain-Barré syndrome (71%)^*^ Crohn's disease (66%)^*^ Hashimoto's thyroiditis (59%)^*^ T1DM (56%)^*^ Scarlet fever (52%)^*^ Idiopathic thrombocytopenic purpura (51%)^*^ Ulcerative colitis (41%)^*^ Multiple sclerosis (41%)^*^ Psoriasis (32%)^*^ Encephalitis or meningitis (591%)^#^ Rheumatoid arthritis (342%)^#^ Rheumatic fever (334%)^#^ Scarlet fever (149%)^#^	Ankylosing spondylitis (178%)^*^ Autoimmune thyroid disease (153%)^*^ Ulcerative colitis (131%; evidence of stronger association in females)^*^ Crohn's disease (44% in females only; negative association in males)^*^ Psoriasis (57% in females, 31% in males)^*^
**Allergies** (% refer to the increase in risk of diagnosis of the specific allergic illness)	Conjunctivitis (33%)^*^ Rhinitis (118%)^*^ Asthma (82%-161%)^*^^#^ Atopic dermatitis (61%)^*^	Higher frequency of eczema^#^	Rhinitis (52%)^¶^ Asthma (34–80%)^¶^ Eczema (32%)^¶^

* *Population-based studies*.

# *Clinic-based studies*.

¶ *Meta-analysis of studies with different design*.

On the other hand, an association between TS and allergies is supported by a more conspicuous body of evidence. Retrospective, case-control population studies of Taiwanese national health insurance datasets (150, 151) reported higher risk of conjunctivitis, rhinitis, asthma, and atopic dermatitis in individuals diagnosed with TS. A higher age- and sex-adjusted comorbidity rate for asthma in TS was also documented through the Canadian Community Health Survey (152). Administering a structured questionnaire to a clinic-based sample of 32 TS patients, Yuce et al. (153) found that more than half of these children presented with allergies, rhinitis being the most common. Evidence of a genetic basis for this comorbidity is increasing, as reported in the *Genetic predisposition* section of this review. Moreover, a predisposition to dysfunctional histamine receptor-mediated signaling in TS suggests a link between type I IgE-mediated hypersensitivity mechanisms and tic generation (154). At the same time, exposure to stressors may facilitate both a surge of tic severity and a flare-up of allergies. New studies should appraise to what degree the association between TS and allergies is supported by concurrent anxiety or depressive disorders, also known to be associated with allergies. Finally, a negative effect on tic severity of anti-allergic pharmacological treatments, steroids in particular, has not been ruled out definitively (155).

### OCD

A potential association between OCD and autoimmune diseases was summarized in the systematic review by Pérez-Vigil et al. (149), which included 74 studies on patients with OCD across all age groups. OCS were confirmed to be common manifestations of Sydenham's chorea. A possible association between a diagnosis of OCD/OCS and a diagnosis of multiple sclerosis or systemic lupus erythematosus was observed, but firm conclusions were precluded by methodological limitations of the included studies, e.g., sample size limitations, involvement of unblinded assessors, use of sub-optimal self-report measures, and lack of control groups. In a Swedish population-based cohort study (36), individuals with OCD were 43% more likely to have any comorbid autoimmune disease compared to individuals without OCD. The increase in comorbidity rate was strongest for Sjogren's syndrome, followed by celiac disease, Guillain-Barré syndrome, Crohn's disease, Hashimoto's thyroiditis, type 1 diabetes mellitus, scarlet fever, idiopathic thrombocytopenic purpura, ulcerative colitis, multiple sclerosis, and psoriasis (Table 2). This study could not confirm the association with Sydenham's chorea due to power limitations. A potential limitation of this work is the likely under-representation of OCD patients who do not seek medical help. In relation to this, Westwell-Roper et al. (72) published the largest to date clinic-based survey that estimated the lifetime prevalence of immune-related diseases using self-report medical questionnaires from 1,401 youth with OCD and 1,045 of their first-degree relatives enrolled in the OCD Collaborative Genetics Association Study. This descriptive study was limited by the lack of a control population, reliance on retrospective self-report and *post-hoc* evaluation of questionnaires that did not screen for all autoimmune comorbidities. Nevertheless, it reported a higher than expected prevalence of scarlet fever, rheumatic fever, rheumatoid arthritis, encephalitis or meningitis in both probands and relatives, independent of OCD status, but not of other immunological disorders. The latter finding is probably due to power limitations and the well-known rarity of autoimmune conditions in children. The reasons of the observed association between OCD and post-streptococcal illnesses should be searched also in shared genetic and/or environmental factors, as well as in dysregulation of mucosal immunity involving the oropharynx.

There is very limited evidence of a relationship between OCD and allergies. A small clinic-based study reported greater frequency of positive skin prick tests in 26 OCD patients compared to control subjects, but not greater eosinophil counts of IgE levels (153). In this study, eczema was significantly over-represented in OCD.

### ADHD

The past decade has consolidated the notion that ADHD is characterized by comorbidity with allergic and autoimmune illnesses. Cross-sectional and longitudinal studies based on clinical services and population health registries have been systematically reviewed and meta-analyzed by two independent groups. In their systematic review, Schans et al. (156) observed that most of the selected studies reported a statistically significant positive association between atopic diseases and ADHD in pediatric populations, suggesting that atopic subjects have a 30–50% greater likelihood of receiving a diagnosis of ADHD. Following meta-analyses of nine selected studies, these authors reported an overall weighted odds ratio of 1.34 (95% CI 1.24–1.44) for asthma, of 1.32 (95% CI 1.20–1.45) for atopic eczema, and of 1.52 (95% CI 1.43–1.63) for allergic rhinitis. Interestingly, they noticed that study heterogeneity was low for the association with eczema and asthma, but substantial for rhinitis. In their systematic review on more than 61,000 children (about 8,000 of whom were ADHD patients), Miyazaki et al. (157) selected a lower number of studies (n = 5), despite a similar methodological design to the review by Schans et al. apart from an apparently more restrictive study selection criteria for allergy and ADHD ascertainment. Overall, the quality of the evidence was rated as low, yet confirming an 80% increase in risk for ADHD in children with asthma. They also highlighted a greater study heterogeneity for the association between ADHD and rhinitis, dermatitis and conjunctivitis. Finally, they reported lack of association between food allergy and ADHD. In line with the evidence summarized above, one study has interestingly reported that greater numbers of atopic comorbidities are significantly related to a greater risk of developing ADHD (158). Individuals with a dual diagnosis of ADHD and chronic tics exhibited a stronger association with the coexistence of multiple allergic illnesses than patients with ADHD without tics (159). Following the two metaanalyses summarized above, Chang et al. (160) used the Taiwan National Health Insurance Research Database to report a higher risk of developing asthma, atopic dermatitis, allergic rhinitis, and allergic conjunctivitis among 20,170 unaffected siblings of ADHD patients compared to >80,000 matched control individuals, with an overall risk increase ranging between 10 and 19%. The relationship between ADHD and allergies is likely to be complex and multifactorial. ADHD has been related to general hypersensitivity to environmental stimuli (161), whereas other authors support a mediational role of inflammatory cytokines and stress to explain this association (143). Larger prospective cohort studies will shed more light on the complex link between allergies and ADHD.

Population-based cohort studies across all age groups, leveraging on the richness of national health registries in Taiwan, Denmark and Norway, have recently provided support for increased prevalence of autoimmune diseases in ADHD. Comparing 8,201 individuals with ADHD to > 36,000 age- and gender-matched control individuals from the Taiwan National Health Insurance Research Database (162), ADHD patients exhibited greater prevalence of ankylosing spondylitis (OR = 2.78), ulcerative colitis (OR = 2.31), and autoimmune thyroid disease (OR = 2.53). Another population-based cohort study of almost one million individuals (>23,000 of whom diagnosed with ADHD) using Danish National Health Registers (38) identified an incidence rate ratio of 1.24 (95% CI 1.10–1.40) for autoimmune diseases in ADHD individuals compared to non-ADHD individuals. The associations between autoimmune diseases and ADHD may be sex-specific. Using Norwegian national registries, Hegvik et al. (163) conducted a cross-sectional study of a cohort of more than 2.5 million individuals, in which the authors could demonstrate sex-based associations between diagnosis of ADHD and different autoimmune diseases. Increased odds for psoriasis were seen in both females and males with ADHD, although the adjusted odds ratio (aOR) was higher in females (1.57 vs. 1.31), with a highly significant interaction of this association with sex. For inflammatory bowel diseases, the association was either observed only in females (ulcerative colitis) or found to be positive in females and negative in males (Crohn's disease). The lack of association with other common autoimmune diseases may depend also on the limited capacity of this study to capture associations with autoimmune diseases of later onset. The observed effect of sex on the relationship between ADHD and autoimmune diseases is intriguing and could be related to sex-specific developmental patterns of immune and neural functioning, as commented in the last section of this review. Moreover, genetic variants associated with the modulation of the risk for ADHD and autoimmunity may be pleiotropic, exhibiting sex-specific associations in opposite directions, as shown for inflammatory bowel diseases and ADHD.

## Insight From Animal Models (Table 3)

Compulsive and repetitive behaviors in rodent and non-human primate models including overgrooming, hoarding and perseverative responding are typically attributed face validity for human OCD. Alongside genetic models targeting proteins involved in synaptogenesis and endocrine models expressing oxytocin and estrogen deficiencies (189), several models primarily involving immune-mediated mechanisms have been proposed in the past decade.

**Table 3:** Summary of findings on maternal immune activation models with a behavioral phenotype exhibiting face validity for compulsions, tics, hyperactivity and cognitive abnormalities observed in attention deficit hyperactivity disorder.

**Type of immunogenic exposure**	**Species**	**Stage of developmental period**	**Behavioral phenotype with face validity for TS, OCD, or ADHD**	**Pathological and other findings**
**TLR4 agonists (LPS)**				
Intraperitoneal single injection of LPS 100 μg/kg (164)	Wistar rats	GD 9.5 (intermediate gestational period)	Increased number of stereotyped movement episodes (grooming)	Increased protein levels of neuron-specific enolase in hippocampus and decreased levels of TGF-β
Intraperitoneal single injection of LPS 100 μg/kg (165)	Wistar rats	GD 9.5 (intermediate gestational period)	Increased frequency and time of head washing episodes and total self-grooming	Dopaminergic hypoactivity decreased levels of homovanillic acid [HVA, dopamine metabolite] and dopaminergic turnover rate
Subcutaneous injection of LPS 500 μg/kg every other day (166)	Wistar rats	From GD 14 to 20 (late gestational period)	Impaired performance in the neurogenesis-dependent novel object recognition test	Persistent microglial activation and downregulated expression of TGFβ_1_ in adult hippocampus and decrease in neurogenesis of the dentate gyrus
Intraperitoneal injection of LPS 50 or 25 μg/kg (167)	CD-1 mice	GD 15-17 (late gestational period)	Progressive age-related decline of spatial learning and memory on the six-radial arm water maze (3–22 months of age)	Decreased levels of histone acetylation and syntaxin-1 and increased levels of synaptotagmin-1 in the dorsal hippocampus
Intraperitoneal injection of LPS 250, 100, or 50 μg/kg (168)	C57Bl/6JOlaHsd mice	GD 15-17 (late gestational period)	Reduced performance in a T-maze learning task	Pro-inflammatory response in fetal microglia, increased proinflammatory activation and decreased BDNF of hippocampal microglia in response to re-challenge with LPS
Intraperitoneal injection of LPS 120 μg/kg (169)	C57BL/6J mice	GD 17 (late gestational period)	Failure to discriminate the novel arm from the familiar one on Y-maze test when exposed also to dietary n-3 PUFA deficiency and deficits in novel object recognition test (spatial memory)	Increased expression of IL-6, TNFα, IL-1β, and IL-10; decreased cFos expression in the dentate gyrus (revealed by dietary n-3 PUFA deficiency)
Intraperitoneal injection of LPS 50 μg/kg (170)	C57BL/6 mice	Postnatal days 3 and 5	Spatial cognitive impairment on the Morris Water Maze	Dysregulated H4K12 (histone) acetylation and impaired c-Fos gene expression in the hippocampus following training
**TLR4 agonists (LPS)**				
Intravenous injection of Poly-I:C (5 mg/kg) (171)	C57BL/6 mice	GD 17 (late gestational period)	Alterations in the locomotor and stereotyped behavioral responses to acute apomorphine treatment in both sexes. Impaired attentional shifting (abnormally enhanced latent inhibition effect) in males	Sex-specific reduction in dopamine, glutamate, GABA and glycine contents in the prefrontal cortex and hippocampus
Intravenous injection of Poly-I:C (2 mg/kg) (172)	*Nurr1*-deficient mice	GD 17 (late gestational period)	Additive MIA and genetic effects on increased spontaneous locomotor hyperactivity in the open field test, disruption of pre-pulse inhibition of the acoustic startle reflex, abnormally enhanced latent inhibition effect, and defective sustained visual attention. Independent effects of MIA on working memory deficits	Synergistic MIA/genetic effects in the disruption of D_2_R expression in the core and shell subregions of the nucleus accumbens and in the decrease and increase in tyrosine-hydroxylase and COMT density in the medial prefrontal cortex
Intravenous injection of Poly-I:C (4 mg/ml//kg) (173)	Rats	GD 15 (late gestational period)	Loss of latent inhibition and excessive sensitivity to the activating effects of amphetamine which emerged in a sex- and behavior-specific manner (earlier in males)	Aberrant postnatal brain development of the hippocampus, the striatum, the prefrontal cortex and lateral ventricles (delayed onset of pathology in females)
Intravenous injection of Poly-I:C (8 mg/kg) (174)	Harlan Sprague-Dawley rats	GD 14 (intermediate gestational period)	Decrease in the rate of route-based learning when visible cues were unavailable in the Cincinnati water maze and reduced prepulse inhibition of acoustic startle in females, but not males; excessive sensitivity to the activating effects of amphetamine	
Intraperitoneal injection of Poly-I:C (5 mg/kg) (175)	CD1 mice	GD 9.5 (intermediate gestational period)	Increased head-twitch response induced by the 5-HT_2A_ receptor agonist DOI in adult offspring mice	Increased density of 5-HT_2A_ receptors in the frontal cortex of adult offspring mice
Intranasal inoculation of Influenza A H3N2 virus (different doses) (176)	BALB/c mice	GD 9 (intermediate gestational period)	Dose-dependent alterations in social and aggressive behaviors in male and female offspring and increases in locomotor behaviors particularly in male offspring	Reduced oxytocin and serotonin levels in male and female offspring and sex-specific changes in dopamine metabolism. Changes in catecholaminergic and microglia density in brainstem tissues of males offspring only
Subcutaneous injection of Poly-I:C (2, 4, 8 mg/kg) (177)	Wistar-Hannover rats	GD 9 & 15 (intermediate & late gestational period)	Decreased amphetamine-induced locomotor responses in offspring experiencing weight loss	Induction of the pro-inflammatory cytokines IL-1β, TNF-α, and IL-6 and of the anti-inflammatory cytokine IL-10 in maternal blood and in fetal central nervous system. No difference in microglia activation.
Intravenous injection of Poly-I:C (8 mg/kg) (178)	Harlan Sprague-Dawley rats	GD 17 (late gestational period)	Decreased startle in males and decreased startle and increased prepulse inhibition of acoustic startle in females. Reduced cued conditioned freezing in males. Impaired Morris water maze hidden platform acquisition and probe performance in both sexes.	
Intravenous injection of Poly-I:C (5 mg/kg) (179)	C57/BLJ6 mice	GD 9 & 17 (intermediate & late gestational period)	Partly age-dependent deficits in hippocampus-regulated spatial recognition memory (Y-maze test)	Impaired hippocampal synaptophysin and BDNF expression.
Intravenous injection of Poly-I:C (5 mg/kg) (180)	C57/Bl6 mice	GD 15 (late gestational period)	Increased basal locomotor activity regardless of period of injection	Decreased brain volume, mainly for posterior brain structures
Intravenous injection of Poly-I:C (4 mg/kg) (181)	Sprague-Dawley rats	GD 12.5 (intermediate gestational period)	Impaired prepulse inhibition of the auditory startle reflex despite preserved performance on the signaled probability sustained attention task	
Intraperitoneal injection of Poly-I:C (20 mg/kg) (182)	C57/BLJ6 mice		Increased basal locomotor activity during the early life period (postnatal day 7)	
**TLR2 agonists (Streptococcus)**				
Group A Streptococcus antigen 1.2 mg (183)	Male Lewis rats	Postnatal 5 weeks + 2 boosts after 14 and 28 days	Increased number of stereotyped behaviors (induced grooming). Impaired food manipulation and beam walking	Antibody deposition in the striatum, thalamus, and frontal cortex, and concomitant alterations in dopamine and glutamate levels in cortex and basal ganglia. IgG reacted with tubulin and caused elevated calcium/calmodulin-dependent protein kinase II signaling in SK-N-SH neuronal cells
Purified IgG from GAS rats as obtained in (183, 184)	Male Lewis rats	Postnatal 5, 7 and 9 weeks	Similar changes as in (183) + increased number of marbles buried on the Marble Burying Test	IgG deposits in the striatum of infused rats colocalized with specific brain proteins such as dopamine receptors, the serotonin transporter and other neuronal proteins
Subcutaneous injection of monoclonal anti-streptococcus IgM and IgG2a antibodies 6.25 or 12.5 μg (185)	Balb/c mice	Adulthood	IgM antibodies: dose-dependent increases in repetitive stereotyped movements, including head bobbing, sniffing, and intense grooming	IgM antibodies: increased Fos-like immunoreactivity in regions linked to cortico-striatal projections involved in motor control, including subregions of the caudate, nucleus accumbens, and motor cortex.
Intraperitoneal injection of 100 μL of serotype Ia Group B Streptococcus (GBS), 10^8^-10^9^ CFU (186–188)	Lewis rats	GD 17-19 (late gestational period)	IgG antibodies: increases in ambulatory activity and vertical activity but not stereotypies Increased spontaneous locomotor activity and worse performance on the Rotarod test (forced motor activity) in females (adulthood). Impaired prepulse inhibition of the auditory startle reflex.	Reduction in the thickness of white matter structures, namely the corpus callosum and the external capsule.Male—but not female—fetuses presented increased levels of IL-1β; fetuses from both sexes increased levels of TNF-α

*The table presents exclusively studies that were published between 2010 and 2019*.

*TS, Tourette syndrome; OCD, obsessive-compulsive disorder; ADHD, attention deficit hyperactivity disorder; TLR, toll-like receptor; LPS, lipopolysaccharide; GD, gestation day; TGF-β, transforming growth factor-β; BDNF, brain-derived neurotrophic factor; Ig, immunoglobulin; PUFA, polyunsaturated fatty acids; Poly I:C, Polyinosinic:polycytidylic acid; GABA, gamma-aminobutyric acid; MIA, maternal immune activation; D_2_R, dopamine D_2_ receptor; 5-HT, 5-hydroxytryptamine; DOI, 2,5-dimethoxy-4-iodoamphetamine; TNF, tumor necrosis factor; IL, interleukin; TNFR, tumor necrosis factor receptor; OCS, obsessive-compulsive symptoms; CCL3, Chemokine (C-C motif) ligand 3; CXCL8, chemokine (C-X-C motif) ligand 8; MCP-1, Monocyte Chemoattractant Protein-1; IP10, Interferon gamma-induced protein 10; T_H_, T-helper; NK, Natural Killer; DAT, dopamine transporter; TSPO-V_T_, translocator protein distribution volume; PET, positron emission tomography*.

The first category of these models comprises immunogenic models, e.g., MIA with exposure to LPS, a cell wall component of Gram-negative bacteria, or Polyinosinic:polycytidylic acid (Poly I:C), a synthetic analog of viral double-stranded RNA, or postnatal exposure to GAS antigens (190). Prenatal exposure (mouse) to 100 μg/kg i.p. LPS at gestational day 9 (164, 165) led to increased frequency of overgrooming behaviors or head washing, increased number of buried marbles in the Marble Burying Test (MBT), and increased repetitive behaviors in the Open Field Test, the latter representative of overanxious behavior. In one of these studies, prenatal LPS exposure was associated with increased dopamine levels in the hypothalamus during the adult period (165). Prenatal exposure (mouse) to 5 mg/kg Poly I:C at gestational day 9 (175) led to increased head-twitch responses induced by the 5-hydroxytryptamine (5-HT)_2A/C_ receptor agonist 2,5-dimethoxy-4-iodoamphetamine. Interestingly, the behavioral effects of Poly I:C during gestation could vary depending on the gestational period, as a subsequent study using the same exposure dose i.v. at gestational day 17 (late pregnancy) decreased food hoarding (179). Postnatal exposure (rat) to 1.2 mg s.c. of GAS exposure at 5, 7, and 9 postnatal weeks led to overgrooming and increased number of buried marbles on the MBT (183, 184), the latter behavior being prevented by ampicillin. The exposure of adult BALB/c mice to GAS monoclonal IgM also caused overgrooming and increased head bobbing stereotypies (185). From a mechanistic point of view, post-streptococcal models yielded changes in neurotransmitter systems, including the dopaminergic (increased dopamine levels and D_1_ and D_2_ receptor expression in the medial and prefrontal cortex and entopeduncular nucleus), serotonergic (lower activity and turnover in medial frontal cortex and cerebellum, prevention of the behavioral phenotype with paroxetine), and glutamate/glutamine (higher levels in the striatum). IgG from GAS exposed animals targeted serotonin and dopamine receptors and the serotonin transporter (184).

The second category of immune-mediated models of OCD comprises genetically manipulated models that involve cell types with immune-modulating function, e.g., microglia. One of the key functions of microglia is to survey the tissue microenvironment, orchestrating the reaction to tissue injury through release of pro-inflammatory effector molecules and phagocytosis of apoptotic debris. These microglial functions modulate also synaptic density and refinement. Loss of function of a particular subset of microglia expressing the transcription factor Hoxb8, which accounts for one third of all the brain adult microglia, causes overgrooming behavior and hair pulling leading to coat loss in mice (191). A more recent study has demonstrated that this phenotype may be associated with anxiety-like behavior and enhanced stress response (raised cortisol levels and pupil fight-or-flight response) in *Hoxb8* KO female mice around the onset of sexual maturity (postnatal 6–8th week), but not in *Hoxb8* KO male animals (192). Blocking estrogens with trilostane or ovariectomy could block coat loss, overgrooming, anxiety-like behaviors and enhanced stress response in *Hoxb8* KO female animals, whereas the administration of 17β-estradiol and progesterone in *Hoxb8* KO male animals replicated the anxiogenic phenotype. This fascinating work suggests a direct interaction between this subset of microglia and synaptic developmental trajectories controlled by sex hormones, which might have important effects on the organization of stress responses and, albeit still unexplored, immune responses. The link between microglia and OCD is supported also by the microglia-specific *Grn* KO model, in which the lack of progranulin is associated with increased internalization of synaptic terminals by microglia, particularly in the ventral thalamus, and with excessive grooming at adulthood (193, 194).

Several MIA models are associated with behavioral, cognitive, and neuroanatomical phenotypes that model human ADHD. However, caution is needed when interpreting this evidence due to heterogeneity of study design (195). Two important sources of heterogeneity include the gestational timepoints of the immunological insult, and the environmental setting in which behavioral outcomes are measured (home cage vs. behavioral testing apparatus). Moreover, most of the behavioral outcomes in the offspring were measured in adulthood, as opposed to during the pre- or peri-pubescent period.

The administration of the TLR4 agonist LPS during *late gestation* (gestational days 14–17) to pregnant rodent dams produced a cognitive phenotype characterized by spatial learning impairment assessed using the Morris-Water (196), the Six-Radial Arm Water (167), or the T-Maze (168) and impairment on the neurogenesis-dependent Novel Object Recognition Test for recognition memory (166, 197). These cognitive defects were associated with selective overactivation of hippocampal microglia (166, 168) and could be exacerbated by polyunsaturated fatty acid deficiency (169). Interestingly, a similar model can induce long-term microglial activation and astrogliosis also in the amygdala (198). Finally, LPS injection during the *neonatal period* can also be associated with spatial learning deficits in adulthood (170).

MIA models associated with TLR2 agonists like inactivated Group B Streptococcus have also been found to generate sex-dependent behavioral responses in the offspring with onset around late puberty, whereby male offspring manifest hyposocial, autistic-like behavioral traits and impaired processing of sensory information, and female offspring show hyperlocomotion and social disinhibition (186, 187, 199). This behavioral sexual dimorphism is associated with sexual differences also in innate immune responses (188).

MIA models using viral TLR3 agonists like Poly I:C administered in most studies during *late gestation* have produced similar behavioral and cognitive phenotypes (180, 200), albeit with exceptions (177, 181), and not always associated with morphological or functional changes of microglia (201). Increased hyperactivity, sensorimotor gating deficits, and attentional shifting and sustained attention impairments were reported from a “dual” model in which Poly I:C administration was given to pregnant dams carrying a heterozygous deletion of *Nurr1* (172). This phenotype was associated with multiple neurotransmitter abnormalities in the prefrontal cortex and ventral striatum. Other reports demonstrated offspring sex-specific responses within Poly I:C or influenza A MIA models (171, 174, 178), with greater hyperlocomotion in males (176, 182), and females exhibiting a later onset of prefrontal cortex and hippocampal volume decreases, attentional deficit, and metamphetamine-induced hyperlocomotion (173). Similar male specificity to locomotor hyperactivity was observed also in prenatal stress models (202).

Juvenile spontaneously hypertensive rats, a metabolic/endocrine model of ADHD, show systemically increased inflammatory cytokines associated with decreased medial prefrontal cortical volume and up-regulation of D2 dopamine receptors. These changes tended to normalize during maturation, in conjunction with compensatory elevation of steroid hormones (203).

Finally, proteomics using induced pluripotent stem cells (iPSC)-derived neurons and microglia from individuals with TS and related disorders will continue to push the field toward better understanding of disease pathophysiology which hopefully will contribute to the future development of therapeutics against specific targets (204, 205).

## Association With Specific Gut Microbiota Profiles

Evidence for a role of the microbiota-gut-brain axis in neural development and behavior is constantly increasing, and has been extensively reviewed (206–208). The influence of symbiotic microorganisms could start even *in utero* via penetration of maternal microbial molecules through the placental barrier, as recent reviewed by Ganal-Vonarburg et al. (209). Immune-based ASD rodent models display imbalanced gut microbiota composition (dysbiosis) and gut barrier dysfunction (“leaky gut”), associated with abnormal social behavior (210, 211). Microbiota composition dysbiosis remains, however, underinvestigated in ADHD and fundamentally unexplored in TS and OCD.

To date, most of the studies that explored the gut microbiome in ADHD have resorted to 16S rRNA amplicon sequencing. This methodological approach allows a snapshot of the taxonomic composition of intestinal microbial colonies (212). However, there is large heterogeneity across studies with respect to sample size and adjustment for several confounding factors, particularly exposure to stimulants, dietary habits, exposure to pro- and antibiotics, and perinatal factors (delivery, early feeding). Moreover, the presence of gastrointestinal dysfunction, which is strongly associated with gut microbiota composition, remains uncertain in ADHD. Index measures of gut microbiota diversity provided discrepant results (213–215). Despite these discrepancies, some differences between ADHD and normotypical youth with respect to relative abundance of gut flora at a genus level have been replicated. Among these, the decreased abundance in *Faecalibacterium* spp. yielded also a negative correlation with ADHD severity scores (215, 216). This genus is associated with anti-inflammatory skewing of immune responses and its decreased abundance is observed in allergies. However, this finding needs to be verified adjusting for dietary habits, as certain diets like the long-term Mediterranean are associated with higher abundance in *Faecalibacterium* spp. Increased abundance in the *Bifidobacterium* genus was found in adolescents/adults with ADHD (217) and, although remaining a controversial finding (218), was reversed by micronutrient supplementation in a recent randomized controlled trial (RCT) (219). The largest study that used 16S rRNA amplicon sequencing investigated adolescents/adults with ADHD and identified increased relative abundance of different, potentially interacting, *Ruminococcaceae* genera, and positive correlation with inattentive symptoms (218). The same group performed an exploratory fecal matter transplantation study of stool specimens from ADHD patients into germ-free mice, generating anxiety-like behaviors (220).

Next generation “shotgun” (metagenomic) sequencing has been applied to a small study of ADHD youth (216). Interestingly, pathway analyses of gene functional annotations revealed abnormalities of metabolic pathways associated with dopaminergic and serotonergic systems, consistent with their hypofunctionality. Additional indirect evidence supporting a role of microbiota in ADHD comes from the observation of greater odds of ADHD among boys with prenatal exposure to dog pets (221), known to influence microbiota composition within familial clusters. Moreover, Slykerman et al. (222) reported an increased risk of ADHD in children exposed to antibiotics in the first 6 months of life, which may represent a surrogate of early infectious exposure, but also a source of gut microbiota imbalance.

The investigation of gut microbiota ecology and its association with pathways modulating the neural-immune crosstalk during development is in its dawning age in the context of TS and OCD. This influence was investigated only in PANS/PANDAS, applying conventional 16S rRNA-based gut microbiome metagenomics to a cohort of 30 patients and a control population. This study revealed gut dysbiosis characterized by an increased abundance of *Bacteroidetes* especially in younger patients, with a negative correlation between genera of the *Firmicutes* phylum and anti-streptolysin O titers (223). Pharmacological animal models expressing compulsive behaviors revealed an association between the behavioral phenotype and changes in gut microbial communities. Chronic quinpirole injections in rats induced locomotor sensitization and compulsive checking associated with changes in abundance of *Firmicutes*, predominantly *Lachnospiraceae* and *Ruminococcaceae* (224). Another study demonstrated different gut microbiota composition between the naturally occurring obsessive-compulsive and the normal phenotypes of deer mice (225). A systematic investigation of the gut microenvironment in patients with OCD or TS has not been published yet. Interestingly, a RCT of probiotic treatment with Lactobacillus and Bifidobacterium species in adults with OCD is currently ongoing (https://clinicaltrials.gov/ct2/show/NCT02334644).

## Responsiveness to Immune-Based Therapies

The therapeutic efficacy of immune-modulatory and anti-inflammatory treatments in TS, OCD, and ADHD has been interrogated by a small number of studies. The use of non-specific immune-modifying approaches such as intravenous immunoglobulins (IVIg) and plasma exchange in the PANS/PANDAS group is supported only by one underpowered RCT as well as uncontrolled case series (226). However, previous attempts to use IVIg in an unselected population of TS patients has not been beneficial, whereas evidence of the efficacy of IVIg and plasma exchange in unselected clinical samples of OCD and ADHD patients is lacking.

A handful of studies have documented the potential effectiveness of anti-inflammatory and antibiotic drugs in decreasing OCS. None of these studies has, however, explored the actual mechanism underlying this potential therapeutic effect. A RCT of 50 outpatients with moderate to severe OCD underwent 10 weeks of treatment with either celecoxib (200 mg bid) or placebo as adjuvant to fluvoxamine (227). A significant effect for “time x treatment” interaction on the Y-BOCS total scores [*F*_(1.38, 66.34)_ = 6.91, *p* = 0.005] was reported, consistent with a more rapid response to celecoxib group than to placebo group (*p* < 0.001), and no difference in adverse events between the two arms.

A small RCT compared cefdinir, a beta-lactam antibiotic promoting glutamate transporter GLT1 expression and enhancing complement-mediated immunity, to placebo over the course of 30 days in 20 subjects with recent onset OCD and/or tics (228). Only a trend was observed for tic severity improvement (44.4% showing at least a 25% reduction in YGTSS), whereas there was no significant difference in OCS severity despite a slightly larger decrease on the CY-BOCS score with cefdinir. This study was underpowered to detect clinically relevant and statistically significant severity changes for both types of symptoms.

A more recent study randomized 102 patients with moderate-to-severe OCD (medication-free for 6 weeks prior to the study) to receive minocycline 100 mg twice per day or placebo for 10 weeks in addition to fluvoxamine 100 mg/day for the first 4 weeks and then 200 mg/day for the rest of the trial (229). There was a significantly greater rate (31.9%) of partial and complete responses in the minocycline group (>35% reduction in Y-BOCS score, *p* < 0.001), with similar frequency of adverse events. Apart from exerting neuroprotective effects decreasing glutamate-induced neurotoxicity, minocycline regulates nitric oxide, TNFα and IL-1β release and showed some benefit in improving schizophrenia, depressive and autistic symptoms. A previous open-label trial by Rodriguez et al. (230) showed that minocycline augmentation may not improve OCD in all adult OCD patients, but only in those with early onset OCD (45%) and with primary hoarding.

Potentially interesting immunotherapy approaches include antibodies or antibody components targeting specific cytokines, such as IL-6, as these may exert relevant effects on neural/immune crosstalks hindering cytokine peripheral receptors from crossing the blood-brain barrier and dampening inflammatory cascades in the periphery.

## Pathophysiological Considerations on Immune Dysregulation in TS, OCD, and ADHD and Questions for Future Research

MIA has become a prominent pathophysiological model for a large series of neurodevelopmental disorders, including ASD, schizophrenia, and bipolar disorder. Even if supported by a substantially smaller body of evidence, it is becoming increasingly convincing that MIA could act as an early hit and a “disease primer” also in the development of OCD, ADHD, and probably also TS. The advancing field of iPSC-derived cellular models has fostered progress toward the development of 3D-brain organoids, which may shed important light on physiological early brain development and basic mechanisms of neurodevelopment-related disorders (205). Interestingly, an organoid model of dorsal forebrain exhibited an organization of cell types that recapitulated in part the developmental trajectory and circuit functionality of the developing human brain (231). This field might offer unprecedented insight into the physiology and pathophysiology of neural network formation (232), including the role of microglia on synaptic formation and refining, as well as on gene-environment interactions during brain development.

Infectious MIA models (LPS- or poly [I:C]-induced) are associated with increased rate of repetitive behaviors modeling compulsions (e.g., overgrooming, head washing), complex tics (head twitch responses secondary to 5-HT_2A/C_ receptor agonists), and hyperactivity/anxious behavior. In a proportion of these studies, the *gestational period* of the immune insult and the *sex* of the offspring are important mediators. With respect to *cognitive phenotype*, spatial exploratory changes were observed following mid-gestational (day 9) exposure to MIA in rodent models, whereas learning changes and dopamine-derived motor behavioral changes have been linked to late (day 16–17) gestational insults. With respect to *repetitive behaviors*, on the other hand, compulsion-like and tic-like behaviors were observed more frequently after mid-gestational immune activation, whereas hyperactivity and over-anxious behaviors could be provoked mostly by late gestational immunological insults. More exploration is necessary to understand the exact immunological pathways that mediate the effect of these insults on synaptic formation, synaptic pruning, and neural migration at different gestational periods. Moreover, we are only beginning the understand the correlation between cognitive/behavioral phenotypes and gestational timing of the insult. Greater clarity will be achieved overcoming the marked methodological heterogeneity across MIA experimental protocols exhibited by studies to date (233). Likewise, there are initial signals of sex-dependent responses in the offspring, which differ in age of onset during postnatal life (older in female offspring), behavioral phenotype (e.g., higher frequency of hyperlocomotion behavior in female offspring), and even maturation of innate immune responses.

The mechanisms through which MIA primes behavior through synaptic refinement of developing neural networks, as well as the development of immune responses, are still under investigation. At a molecular level, the production of pro-inflammatory cytokines like IL-1β, TNF-α, IL-17-α, and IL-6 has classically been considered as a major culprit of region-specific neuropathologic alterations producing long-lasting effects in adult offspring. Although these cytokines may be produced by different neural cell types, including neurons and astrocytes, their production by microglia, alongside growth factors and oxidative stress markers, has emerged as pivotal in the genesis of neural and immune abnormalities linked to different neurodevelopmental disorders. Microglia can respond to MIA insults even since its neural cell progenitor stage of development (yolk sac macrophage stage) around day 10 of gestation. Remarkably, immunological priming of early microglia can set transcriptomic signatures in these cells that may last until adulthood. It is therefore intriguing to hypothesize that the transcriptomic profile detected *post mortem* in brains of TS patients (109), or the activated microglial pattern observed using PET in young adults with OCD (132), might have been predetermined, at least in part, already since the intrauterine period. The initial evidence in favor of an age-dependent variability of MIA effects in the offspring can also be explained with the highly dynamic functional pattern of microglial activity during the course of development (234, 235). The central role of microglia in abnormal neural-immune development in the context of OCD is strongly supported by the overgrooming phenotype exhibited by mice lacking one of the most abundant sets of microglia (*Hoxb8*-positive), or by mice lacking the microglia-specific *Grn* gene. The first of these two transgenic models provides further support to the estrogen-dependence of microglial effects on the development of overgrooming, anxiety-like behaviors and enhanced stress responses. This sexual dimorphism is of exceptional interest, as it may point to sex-specific mechanisms in the microglial modulation of neural-immune developmental abnormalities in OCD and related conditions. The maturation and functional activation of microglial populations follow sex-determined neurodevelopmental patterns. Sexually dimorphic responses to immune-activating triggers (e.g., Poly I:C) are recognized with respect to microglial density, morphology, and transcriptional profile. Moreover, this sexual dimorphism appears to be long-lasting, as the female offspring of MIA-associated pregnancies display greater microglial activation in adulthood compared to male offspring. This is intriguing at the light of an aging-progressive decrease of the male predominance in the sex ratio observed in both TS and OCD. Finally, there is limited evidence of dual-hit processes associated with MIA in ADHD, TS and OCD. An exception is the interaction between MIA and *Nurr1* genotype demonstrated in a poly(I:C) model of ADHD, with associated abnormalities in the prefronto-striatal pathways.

At difference from infectious MIA models, the effect of non-infectious causes of MIA (prenatal stress, maternal diet/obesity, microbiome) await to be investigated in the context of TS, OCD, and ADHD. Microglial activation, with changes in morphology and cytokine production, and migration of GABAergic progenitor migration were reported in the offspring of prenatal stress (restraint/bright light) MIA models of ASD (236), with predilection for the female offspring. The effect of maternal diet and obesity has also been explored in the context of an ASD-like phenotype, and linked to decrease in oxytocin-producing cells in the paraventricular nucleus and decreased synaptic plasticity in the ventral tegmental area (237).

Human studies offer limited information on the role of MIA in the pathogenesis of these neurodevelopmental disorders. The association between infections during pregnancy remains unexplored in TS and OCD, whereas data on ADHD carry known limitations coming from national registry datasets, first of all the lack of serial serologic measurements to prevent biases in the ascertainment of the exposure. The lack of birth seasonality in OCD, TS, and ADHD indirectly does not speak in favor of an effect of maternal infection on the risk for these disorders in the offspring (238). The effect of stressors in pregnancy also remains underexplored in these conditions. There is, nevertheless, well-established evidence that stressors cause overproduction of proinflammatory cytokines by immune cells in response to immunostimulants, and alterations in the HPA axis, which may also influence the development of immune responses in the offspring, especially T and B cell proliferation, NK cell cytotoxicity, and cytokine receptor density (239, 240).

The involvement of mechanisms occurring at the placental interface in the pathoogenesis of TS, OCD and ADHD also remains unexplored. MIA models based on Poly I:C demonstrated the activity of maternal immune cells in the placenta, particularly in association with increased IL-6 production at the maternal-fetal interface. The placenta might also modulate stress responses induced by prenatal stress-induced MIA, mediating the metabolism of glucocorticoids into inactive metabolites (240).

Human studies that explored the physiological profile of immune-inflammatory responses and the clinical profile of immunological comorbidities in patients with TS, OCD, and ADHD consistently indicate the long-term persistence of abnormally enhanced responses to exogenous (pathogens, allergens) and endogenous (self-antigens targeted by autoimmune processes) triggers. The limited evidence of dysregulated expression of immune effector molecules (e.g., cytokines) in individuals with established ADHD is at odds with the robust evidence of comorbidity of allergies and autoimmune diseases in this condition, and is probably due to the lack of adequately powered clinical observational studies. An abnormal immune priming, probably acting on a combination of predisposing genomic factors and possibly promoted by epigenetic modifications, may contribute to altering the process of maturation of specific neural networks, as well as of both innate and adaptive immune regulatory mechanisms. The resulting outcome of this pathophysiological substrate is likely to be the long-lasting co-existence of behavioral and cognitive deficits *and* hypersensitive innate and adaptive responses to antigens or allergens of sufficient immunogenic potency. Our critical review of the literature suggests that there is insufficient evidence to support the hypothesis that immunogenic triggers cause or predict the short- or medium-term worsening of tics, OCS, attention/executive deficits, or hyperactivity/impulsivity. In the case of tics, there is actually mounting evidence supporting the lack of association between common immunogenic triggers e.g., GAS infections and exacerbations of tics. Alternatively, it seems more likely that enhanced immune-inflammatory responses toward pathogens could be epiphenomenal to tics and related symptoms, as an increase in immune responses targeting GAS and other pathogens appears as longstanding in these patients (64, 241). As mentioned above, this could stem from a vulnerability deriving from genetic background and abnormal immune priming in sensitive developmental periods. At the same time, the contribution of a “reverse causation” mechanism, whereby behavioral patterns that are especially characteristic of the ADHD spectrum could increase the likelihood of contacts with pathogens or exposure to certain allergens.

The involvement of the microbiota-gut-brain axis in the neuroimmune connection that influences network maturation trajectories in TS, OCD, and ADHD is still unchartered territory that awaits to be explored. The limited evidence we have available today suggests the presence of a few differences in relative abundance of microbiota constituents between ADHD and neurotypical individuals, and a possible influence on neurotransmitter pathways of the microbiome profiling. Future research should employ germ-free versions of animal models of TS, OCD, and ADHD in order to investigate the contribution of the microbiome to sexually dimorphic characteristics of neural development and maturation of microglia in these disorders. It would also be very interesting to explore the production of bacterial metabolites that are known to steer microglial function, e.g., short chain fatty acids and tryptophan-derived ligands, across different age groups in patients with TS, OCD and ADHD. Metagenomic and metabolomic studies related to the microbiome profiling are warranted to discover the most relevant metabolic pathways influenced by gut microbiota in these disorders, and design mechanism-driven microbiota manipulation interventions.

Behavioral therapies based on habit reversal training or exposure-response prevention constitute first-line interventions in the management of tics and obsessive-compulsive symptoms. However, little is known on their potential impact on the regulation of immune responses in patients with TS and OCD. There is increasing evidence suggesting an effect of cognitive-behavioral therapy (CBT) on immune markers in other psychiatric conditions. In major depressive disorder (MDD), a greater reduction of pro-inflammatory markers (TLR-4 RNA and protein and NF-κβ RNA) during CBT was associated with greater clinical improvement (242). Moreover, baseline levels of pro-inflammatory cytokines (IL-12, IL-1β) may predict the response of depressive symptoms to CBT in youth with bipolar disorder or MDD (243). Interestingly, a similar effect of decreased plasma chemokine levels was reported for internet-based CBT in patients with mild-moderate depression (244). Finally, other psychotherapeutic approaches still under-investigated in these conditions, e.g., mindfulness meditation, appear promising for the modulation of inflammation and cell-mediated immunity (245). It would be interesting to explore whether immune-based mechanisms contribute to the efficacy of comprehensive-behavioral intervention for tics (CBIT) or ERP in TS and OCD patients.

## Closing Remarks

Neural development is an enormously complex and dynamic process. Immunological pathways are intimately involved in several very early neurodevelopmental processes including the formation and refinement of neural circuits. Hyper-reactivity of systemic immune pathways and neuroinflammation may contribute to the natural fluctuations of the core behavioral features of tic disorders and related conditions including OCD and ADHD (Box 1). While the genetic relationship amongst these disorders is complex, and potentially linked to specific sub-phenotypes, it is clear that early environmental risk factors can influence the course and severity of these disorders (Box 1). Future research is needed to gain a deeper understanding of these gene-environment interactions and how they set the stage from a neurodevelopmental perspective for the emergence of these disorders. which may explain differences in the strength of their pairwise genetic correlation (Box 2). Likewise, future research also needs to focus on the key molecular pathways through which dysbiosis of different tissue microbiota influence neuroimmune interactions in these disorders, and how microbiota modification could impact their natural history. Efforts need to be made to establish valid biomarkers that will guide a personalized approach to the treatment of these conditions.

Box 1 **Summary key points on the relationship between immune-related mechanisms and Tourette syndrome (TS), obsessive-compulsive disorder (OCD), and attention deficit hyperactivity disorder (ADHD).**

- Initial research demonstrates an association between OCD and ADHD and genes that are implicated in immune system function
- Several immune-mediated diseases also have genetic correlation with TS and ADHD
- The data investigating a potential correlation between postnatal Group A Streptococcus (GAS) or non-streptococcal infections and the development of tic disorders or obsessive-compulsive symptoms remains mixed
- There is evidence for an association between significant infectious exposure in early childhood and the development of ADHD symptoms
- Direct evidence of pro-inflammatory overactivity in the brain in TS and OCD is modest and limited
- There is preliminary but compelling evidence to indicate that dysfunction in microglial maturation and functioning is implicated in TS
- There is limited, strong evidence for a correlation between the activation of microglia and OCD
- In younger patients with ADHD, there is modest evidence supporting the overactivity of systemic inflammatory mechanisms
- TS and ADHD have been associated with an increased risk for multiple autoimmune disorders and allergic conditions
- OCD has been associated with an increased risk for multiple autoimmune disorders, but has not been associated with an increased risk for allergic conditions
- Patients with ADHD demonstrate some evidence of alterations in the gut microbiome, whereas this topic has been studied only minimally in TS and OCD
- Direct and indirect immune-modulatory interventions aimed at treating TS, OCD, and ADHD have only been studied to a limited degree
- Maternal immune activation is likely involved in the early development of OCD, ADHD, and probably also TS
- There is insufficient evidence that immunogenic triggers influence short- or medium-term worsening in tic symptoms, obsessive compulsive symptoms, or symptoms of inattention or hyperactivity

Box 2 **Questions that should be addressed by future research projects exploring the relationship between immune-related mechanisms and Tourette syndrome (TS), obsessive-compulsive disorder (OCD), and attention deficit hyperactivity disorder (ADHD).**

- What are the key genetic and epigenetic factors that influence a dysfunctional neural-immune crosstalk during development in TS, OCD, and ADHD?
- Can induced pluripotent stem cells-derived cellular models and 3D-brain organoids help recapitulating the dysfunction of microglia interaction with other neural cell types that may occur during development in individuals with TS, OCD, and ADHD?
- Do sex-specific alterations of microglia development contribute to the sexual dimorphism observed in the pathological manifestations of TS, OCD, and ADHD?
- What are the key molecular pathways by which changes in microbiota play a role in neuroimmune interactions in TS, OCD, and ADHD? Is the development of microglia the critical physiological process influenced by symbiotic microbiota in the pathogenesis of TS, OCD, and ADHD?
- Will immune-modifying treatments targeting specific molecular pathways be able to counteract abnormal neurodevelopmental trajectories in TS, OCD, and ADHD?
- Will microbiota manipulation strategies targeting specific molecular pathways be able to counteract abnormal neurodevelopmental trajectories in TS, OCD, and ADHD?
- Will the analysis of multivariate datasets, incorporating metagenomics, metabolomics, “inflammasomics,” gastrointestinal, and CNS physiomarkers, pay off in accelerating the discovery of key mechanistic pathways to prevent and modify disease course in TS, OCD, and ADHD?

## Author Contributions

DM and JL contributed to conception and design of this review. DM prepared the initial draft of the manuscript. Each of the authors then critically reviewed and revised the manuscript before approving for submission the final version of the article.

## Conflict of Interest

The authors declare that the research was conducted in the absence of any commercial or financial relationships that could be construed as a potential conflict of interest.
